# Adolescent and young adult periodontitis: modifiable inflammatory pathways and risk stratification

**DOI:** 10.3389/fmed.2026.1870444

**Published:** 2026-07-08

**Authors:** Jiangtao Lv, Yan Sheng, Chun Lan, Guohui Bai, Na Zhao, Shuai Wang

**Affiliations:** 1 School and Hospital of Stomatology, Zunyi Medical University, Zunyi, Guizhou, China; 2 Department of Preventive and Pediatric Dentistry, Hospital of Stomatology, Zunyi Medical University, Zunyi, Guizhou, China; 3 Department of Periodontics and Oral Medicine, College of Stomatology, Guangxi Medical University, Nanning, China

**Keywords:** adolescents, diet and behaviors, iatrogenic factors, modifiable risk factors, obesity, periodontitis, plaque retention, tobacco

## Abstract

**Objective:**

This review summarizes current evidence on modifiable risk factors for periodontitis in adolescents and young adults, with a focus on their underlying inflammatory pathways and mechanisms in early disease progression.

**Methods:**

A structured literature search was performed in PubMed/MEDLINE and Web of Science for human studies published from 2000 to 2025, examining gingival and periodontal outcomes in relation to four major modifiable exposure clusters: tobacco use, obesity and metabolic health, diet and related behaviors, and plaque-retentive or iatrogenic factors.

**Results:**

Available evidence indicates that early inflammatory indicators of periodontal risk, particularly bleeding on probing and calculus, are highly prevalent in young populations and closely associated with these modifiable exposures. These factors drive periodontal pathogenesis by increasing inflammatory burden, promoting microbial dysbiosis, impairing host immune and metabolic responses, and accelerating the transition from gingivitis to periodontitis. Importantly, modifiable risks and early periodontal burden are not uniformly distributed across the population, but cluster within distinct high-risk subgroups.

**Conclusions:**

These findings indicates that early periodontitis in youth arises from concentrated risk exposure rather than a population-wide downward shift in age at onset. Targeted, multi-level preventive strategies for high-risk subgroups that address underlying modifiable inflammatory pathways may effectively delay the onset and progression of periodontitis and exert beneficial effects on long-term periodontitis-related systemic health outcomes.

## 1 Introduction

Periodontitis is among the most burdensome chronic inflammatory oral diseases worldwide and can lead to tooth loss, impaired mastication, and diminished quality of life (1). A growing body of evidence suggests bidirectional associations between periodontitis and multiple systemic conditions (e.g., diabetes and cardiovascular disease), with the potential for mutual influence and amplification of disease course (2, 3). Although traditionally regarded as a disorder of mid- to late adulthood, multi-source surveillance data and cohort studies indicate that periodontitis may begin earlier in the life course (4, 5). This implies that timely identification and early intervention for periodontal problems during adolescence and young adulthood may not only help preserve oral health and delay disease progression, but may also have broader implications for subsequent systemic health outcomes and long-term health trajectories. Among adolescents and young adults, early indicators of periodontal inflammation or susceptibility, such as bleeding on probing (BOP) and calculus, are being detected more frequently, and some studies report increased incidence of clinically diagnosed periodontitis in young adulthood (6, 7). These early inflammatory signs reflect heightened inflammatory burden and represent upstream precursors on the pathway to periodontitis. Whether these patterns reflect a true downward shift in the age distribution, or are primarily driven by changes in diagnostic criteria, measurement error, care-seeking behavior, and sampling differences, remains to be clarified by further research.

Under the 2017 World Workshop stage–grade framework, case identification primarily focuses on clinical attachment loss (CAL), with probing pocket depth (PPD) and radiographic bone loss (RBL) employed to stage disease severity and assess the risk of progression (8). Comparisons across time and regions are complicated because many population surveys still rely on the WHO Community Periodontal Index (CPI)—particularly in adolescents, among whom bleeding on probing and calculus are recorded far more often than the attachment loss required for case definition (9). Partial-mouth recording protocols further underestimate the prevalence and severity of periodontitis (10, 11). Use of radiographic assessment varies across surveys, limiting comparability; within the 2017 framework, radiographic bone loss serves as supportive evidence for staging (12), underscoring the need to distinguish gingivitis indicators from confirmed periodontitis. Therefore, throughout this review, gingivitis-related indicators such as BOP, gingival inflammation, plaque, and calculus are interpreted as early risk states rather than established periodontitis, whereas confirmed periodontitis is defined by irreversible periodontal tissue destruction, particularly CAL, with PPD and RBL used as supportive indicators.

Because measurement indicators and modifiable risk factors vary across the different stages from gingivitis to periodontitis, a single PICO-based meta-analysis may not adequately or accurately capture the existing evidence. Therefore, we conducted a structured narrative synthesis based on predefined search concepts and eligibility criteria. This review addresses three main aspects: (1) assessment indicators for early periodontal risk in youth; (2) modifiable risk factors and their underlying inflammatory pathways in this population; (3) the role of early prevention and risk stratification at school, community, and primary care levels. By addressing these aspects, current challenges can be better understood, thereby informing future prevention and intervention strategies.

## 2 Methods

### 2.1 Review design

This review adopted a structured narrative approach following a predefined protocol, including systematic literature searching, study selection and thematic synthesis to ensure transparency and reproducibility. Narrative synthesis was used to integrate evidence across studies with heterogeneous designs, outcome definitions and exposure measurements. All findings were synthesized qualitatively.

### 2.2 Outcome classification

Based on the continuum of periodontitis progression, this review divided disease manifestations into two sequential stages for separate analysis. Line A represents early risk states characterized by initial inflammatory signs, including non-case-defining indicators of gingival inflammation and plaque accumulation, such as BOP, gingival indices, plaque and calculus levels. These markers reflect gingival inflammation or elevated periodontal risk rather than definitive periodontitis (13, 14). Line B represents confirmed periodontitis, defined according to contemporary classification criteria primarily by CAL, supplemented by PPD and RBL where available. This distinction is designed to improve comparability across studies. It covers the full progression continuum of periodontal disease (15, 16), with Line A corresponding to early-stage alterations and Line B representing irreversible tissue damage in advanced stages, thus enabling evidence synthesis throughout the entire disease course and avoiding omission of key disease progression stages (17, 18). This classification was also used to reduce misinterpretation caused by heterogeneous diagnostic criteria across studies and to avoid equating gingivitis-related inflammatory signs with established periodontitis.

### 2.3 Risk-factor clusters and rationale

The risk factors included in this review were categorized into four predefined clusters: (1) tobacco use and electronic cigarette exposure; (2) obesity and metabolic disorders; (3) dietary patterns and related lifestyle behaviors; and (4) plaque-retentive anatomical features and iatrogenic factors. These clusters were defined *a priori* rather than *post hoc*, to align with a prevention-focused life-course framework tailored to adolescents and young adults.

Four core considerations guided this categorization: (1) Biological plausibility: these exposures have well-documented links to periodontal inflammation and disease progression; (2) Population relevance: these risk factors are prevalent or increasingly common among young populations; (3) Modifiability and implementability: they are feasible for targeted intervention in school, community, family and primary care settings; (4) Evidence availability: sufficient human studies report measurable outcomes that can be mapped to Line A and/or Line B. This grouping reflects major prevention-oriented pathogenic pathways covering behavioral, metabolic, dietary, local anatomical and iatrogenic domains, rather than aiming to provide a complete etiological framework. Since periodontitis arises from the combined effects of local and systemic influences, the present classification prioritizes modifiable risk factors with high practical value for public health intervention in adolescents and young adults.

### 2.4 Research strategy

We searched PubMed/MEDLINE and Web of Science for studies published from January 2000 to July 2025. Full search strategies are provided in Supplementary Table S1. Studies were included if they involved adolescents and young adults (or age-stratified data), reported outcomes mappable to Line A and/or Line B, and examined at least one predefined risk exposure. Non-human/*in vitro* studies and reports without relevant periodontal outcomes or interpretable exposure data were excluded. After duplicate removal, titles and abstracts were screened by one author (J.L.) and independently checked by a second author (N.Z.), with disagreements resolved by consensus (S.W., G.B.). This review incorporates elements aligned with structured evidence synthesis, including transparent reporting of search strategy, eligibility criteria, and study selection process. Quantitative synthesis was not performed due to heterogeneity across studies.

## 3 Modifiable inflammatory pathways

### 3.1 Overall epidemiological context

Recent WHO estimates indicate that severe periodontal disease affects more than 1 billion people worldwide (19). Consistent with this, Global Burden of Disease (GBD) assessments show that severe periodontitis has remained a high-burden condition since 1990, with marked regional heterogeneity in trends (including an estimated 1.1 billion people living with severe periodontitis in 2019). Although the overall increase in the absolute number of affected individuals has been driven largely by population aging and growth (20), a life-course perspective suggests that periodontal burden in younger populations is not uniformly distributed (21, 22).

**Figure 1:**
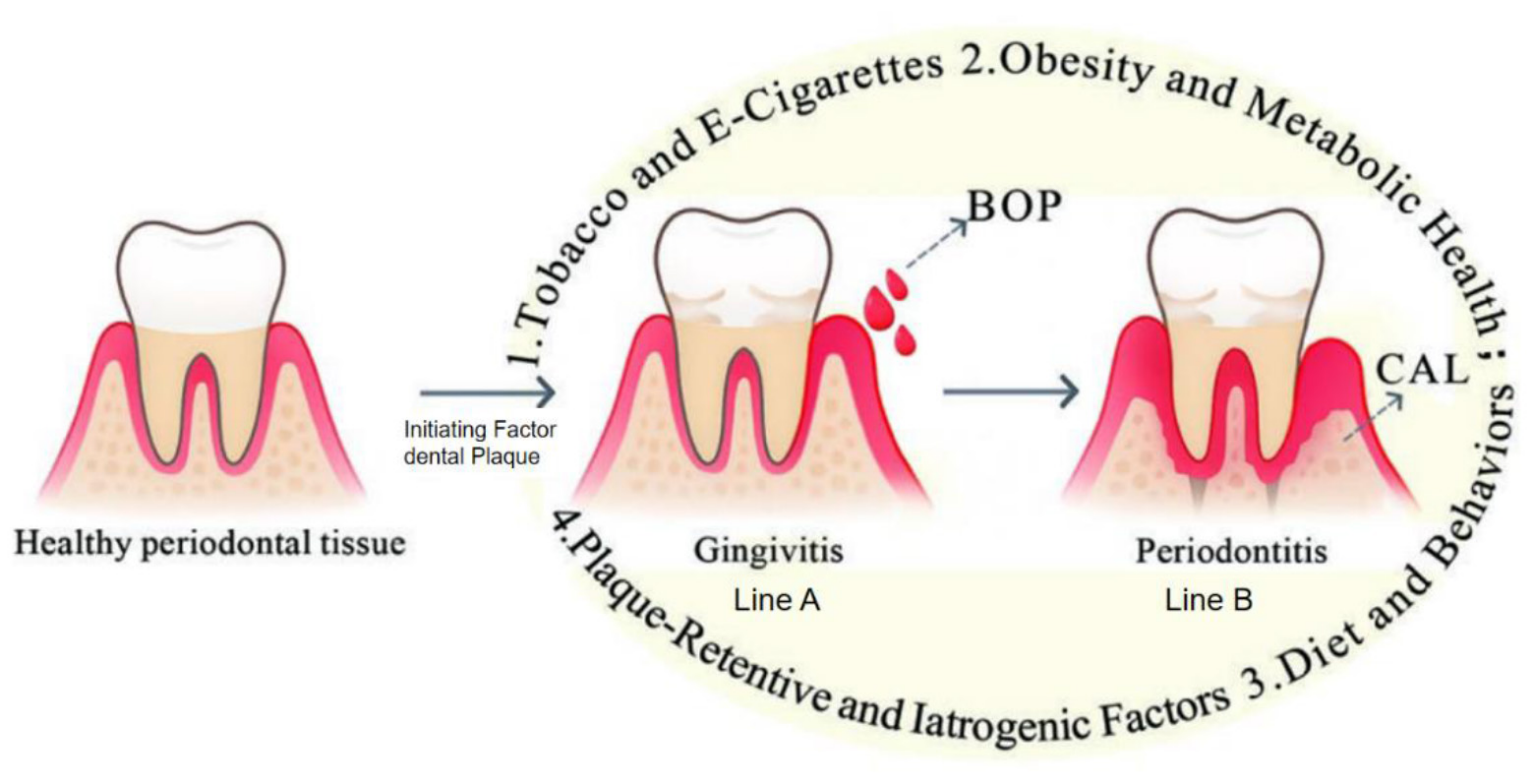
Conceptual framework of Line A and Line B. Progression of periodontitis, initiated by dental plaque, gradually developing into gingivitis (with BOP as its main manifestation), and ultimately progressing to irreversible periodontitis (with CAL as a key marker of the disease), with modifiable risk factors playing a role throughout the process. BOP, bleeding on probing; CAL, clinical attachment loss.

GBD-based and related analyses suggest an increasing incidence in adolescents and young adults in some regions (23). This pattern appears to be concentrated in subgroups with clustered high-risk exposures, including tobacco smoking/e-cigarette use, obesity and metabolic dysregulation, unhealthy diet and oral health behaviors, and plaque-retentive or iatrogenic conditions, which may accelerate progression from reversible gingival inflammation to irreversible attachment loss (24). Consistent with this, youth surveillance commonly reports high levels of Line A indicators, with substantial cross-country variation, and these indicators frequently cluster with adverse behaviors and environmental exposures (25). For example, in Greek adolescents, BOP was reported in 23.6% of 12-year-olds and 21.6% of 15-year-olds, while calculus was observed in 46.2% and 44.3%, respectively (9). In 12-year-old Chinese adolescents in Sichuan (7), gingival bleeding and calculus were reported in 46.63% and 66.94%, respectively. Among Japanese high school students aged 15–18 years, gingival bleeding and calculus were 44.2% and 42.2%, respectively (6). Emerging chairside and salivary biomarkers (e.g., aMMP-8) further suggest that a subset of adolescents may have heightened inflammatory activity even when deep pockets are uncommon (26). Clinical observations also indicate that deep periodontal pockets detected in late adolescence or early adulthood, if untreated, are associated with subsequent periodontal deterioration (4). Taken together, the evidence supports a stratified life-course pattern in which elevated Line A burden identifies vulnerability and exposure accumulation, while increases in Line B burden are more likely to emerge in high-risk subgroups within this population rather than evenly across the population (Figure 1).

### 3.2 Tobacco and e-cigarettes

Tobacco-related exposure is an important modifiable risk factor that may influence the occurrence and development of periodontitis in adolescents and young adults through multiple inflammatory and microbial pathways. Evidence from adult and mixed-age populations consistently shows that combustible tobacco use is associated with poorer periodontal clinical outcomes, whereas smoking cessation may improve the host–biofilm balance and treatment responsiveness (27, 28). However, when applying these findings to adolescents and young adults, age-specific exposure patterns and behavioral characteristics should be considered.

In adolescents and young adults, tobacco and nicotine exposure often begins during a critical developmental period and may occur in patterns that differ from those in older adults. These include early initiation, intermittent or social smoking, experimentation with flavored products, e-cigarette use, and dual use of combustible cigarettes and electronic nicotine delivery systems. Such patterns may result in repeated low-to-moderate nicotine exposure from an early age, even before substantial cumulative periodontal destruction becomes clinically detectable. Among adolescents and young adults, smokers may appear clinically “healthy” yet exhibit a disease-like subgingival ecology and are more prone to adverse periodontal conditions (29). Driven in part by the substantial harms of combustible products, e-cigarette use has risen among youth; nevertheless, emerging evidence indicates that vaping still impairs periodontal tissues (30), including elevated inflammatory markers, a higher proportion of periodontal pockets ≥4 mm, and a distinctive “vaping-associated” subgingival microbiome enriched for pathogens and pro-inflammatory cytokines (31, 32). Table 1 summarizes several studies on the impact of tobacco and e-cigarette (ENDS) use on periodontal health, which further supports the significant role of tobacco and vaping in exacerbating periodontal disease. These studies indicate that both cigarette smoking and e-cigarette use contribute to periodontal breakdown through multiple inflammatory and microbial pathways, including microbial shifts and inflammatory responses. Notably, e-cigarette use, like smoking, is linked to a distinctive microbial profile associated with periodontal disease, even in individuals who may not show visible clinical signs of periodontitis.

**Table 1:** The impact of tobacco and e-cigarette (ENDS) use on periodontal health. The table summarizes studies showing that smoking and vaping lead to periodontal damage through microbial shifts, inflammation, and distinctive subgingival microbiomes, increasing periodontal risk. BOP, bleeding on probing; CAL, clinical attachment loss.

Studies on the impact of tobacco and e-cigarette (ENDS) use on periodontal health						
Study	Study design	Sample & age	Putative mechanism(s)	Key periodontal indicators/outcomes	Applicability to the target age group	PMID
Fullmer et al. (33)	Prospective longitudinal sub study	Smokers with moderate-to-severe chronic periodontitis; n = 22 (11 quitters, 11 continuing smokers); age: NR.	Smoking cessation may improve periodontal health by altering subgingival microbial community composition (mainly relative abundance shifts).	Quitters showed significantly different subgingival microbial profiles vs. continuing smokers at 6–12 months; both groups had PD reduction after therapy, with no between-group differences in PD/plaque scores at sampled sites.	Indirect adult evidence; age not reported; retained as supplementary mechanistic evidence.	19587156
Machuca et al. (34)	Cross-sectional comparative study	*n* = 304 young Caucasian males entering the Armed Forces; mean age 19.38 ± 0.72 y	Cigarette smoking may impair periodontal health via altered inflammatory/vascular response (lower gingival bleeding despite worse periodontal parameters).	Smokers showed lower PBI but deeper PD and higher CAL than non-smokers; tobacco use adversely affected periodontal health even at a young age.	Direct young-adult evidence.	10695941
Mason et al. (29)	Cross-sectional 16S microbiome study (smokers vs. never-smokers).	*n* = 200 periodontally healthy adults (smokers and nonsmokers), 21–40 y.	Smoking may increase periodontitis risk by creating an at-risk subgingival microbiome (pathogen-rich, commensal-poor, anaerobic), reducing health-compatible community stability (“niche saturation”).	Smokers showed distinct subgingival microbial clustering at all taxonomic levels, higher Shannon diversity, and enrichment of periodontal/systemic pathogens with depletion of health-associated commensals, despite clinical periodontal health.	Mixed-age evidence; includes young adults, but applicability to adolescents is limited.	25012901
Xu et al. (32)	Prospective longitudinal clinical study	*n* = 101 periodontitis patients completing follow-up (31 cigarette smokers, 32 e-cigarette users, 38 nonsmokers); age ≥21 y.	E-cigarette use may promote periodontal dysbiosis by enriching periodontal pathogens and increasing proinflammatory cytokines, with microbiome–cytokine correlations supporting an inflammatory microbial shift.	E-cigarette users showed salivary microbiome profiles more similar to cigarette smokers than nonsmokers at the same disease stage, with enrichment of periodontal disease-associated taxa (including Filifactor, Treponema, and Fusobacterium) and increased salivary proinflammatory cytokines over time (notably IFN-γ and TNF-α)	Mixed-age adult evidence; includes young adults, but extrapolation to adolescents requires caution.	34997976
Javed et al. (31)	Cross-sectional pilot clinical study	*n* = 94 males (33 smokers, 31 e-cig users, 30 never-smokers); mean age: 41.3, 37.6, and 40.7 y, respectively.	Smoking/vaping may affect periodontal status via nicotine-related vasoconstriction (masking BOP) and oxidative stress/proinflammatory effects; higher smoking burden may worsen plaque accumulation and pocketing.	Smokers had higher PI and PD ≥4 mm than e-cig users and never-smokers; BOP was higher in never-smokers; no significant differences in MT, clinical AL, or MBL among groups.	Indirect adult evidence; retained as supplementary evidence on vaping-related periodontal parameters.	28644108
Xu et al. (35)	6-month longitudinal clinical study	*n* = 101 periodontitis patients at follow-up (31 smokers, 32 e-cig users, 38 non-smokers); age ≥21 y.	E-cigarette aerosol may alter the oral microbiome and host inflammatory response, promoting dysbiosis and periodontal tissue damage.	BOP and PD increased over time in all groups, but CAL increased only in e-cigarette users; severe periodontitis was more frequent in smokers and e-cigarette users than non-smokers, with age confounding group differences.	Mixed-age adult evidence; includes young adults, but extrapolation to adolescents requires caution.	35048050

The target age group refers to adolescents and young adults. Studies involving broader adult or middle-aged samples were retained only as supplementary mechanistic or contextual evidence and were not considered direct evidence for adolescents and young adults.

Tobacco is associated with periodontal breakdown through multiple pathways: (1) vascular and immune modulation that masks bleeding phenotypes and dampens host defenses (28, 33); (2) neutrophil dysfunction and dysregulated cytokine/growth-factor signaling that amplify systemic and local inflammation (36, 37); (3) impaired fibroblast adhesion and collagen synthesis with activation of matrix metalloproteinases, leading to connective-tissue degradation (38); (4) increased oxidative stress and ecological dysbiosis favoring enrichment of anaerobic pathogens (39); (5) imbalance of osteoclast-related signaling (e.g., RANKL/OPG) that accelerates alveolar bone resorption (40).

Evidence for conventional cigarette smoking is relatively strong, supported by longitudinal, clinical, and mechanistic studies. However, evidence on e-cigarettes remains emerging and is mostly based on cross-sectional or short-term studies, often in adults. Therefore, findings on vaping should be extrapolated to adolescents and young adults with caution.

### 3.3 Obesity and metabolic health

In recent years, the prevalence of overweight and obesity has increased steadily across all age groups, with a notable shift toward adolescents and young adults (41, 42). This trend parallels the rise in metabolic abnormalities, such as insulin resistance (IR), dyslipidemia, impaired glucose tolerance, and metabolic syndrome. A growing body of evidence suggests a bidirectional relationship between obesity/metabolic dysfunction and periodontitis. Systemic metabolic inflammation mediates this mutual influence, exacerbating periodontal breakdown, while chronic periodontal infection contributes to systemic metabolic dysregulation (43, 44).

Epidemiologic studies consistently show that higher adiposity is associated with increased periodontal inflammation and attachment loss, although the magnitude of these effects varies across different populations and outcome measures (Table 2). For instance, in young adults from Brazil, obesity was linked to gingival bleeding and calculus, but not to periodontal pockets (47). Similarly, Japanese university cohorts demonstrated that each 1 kg/m^2^ increase in BMI raised CPI-defined periodontitis risk by approximately 16%, and BMI gain over time—especially without interdental cleaning—worsened bleeding on probing (48, 49). Large-scale national data from the U.S. NHANES confirmed that general and central obesity remained independently associated with higher periodontitis prevalence after multivariable adjustment (50).

**Table 2:** The table summarizes studies on the impact of obesity, metabolic disorders, and diet on youth periodontal health. These factors increase periodontal risk through inflammation, microbiome alterations, and poor hygiene. Obesity is linked to indicators like bleeding on probing and clinical attachment loss.

Evidence on the impact of obesity, metabolic disorders, and diet on periodontal health in youth						
Study	Study design	Sample & age	Putative mechanism(s)	Key periodontal indicators/outcomes	Applicability to the target age group	PMID
Tsai et al. (45) (CHIEF Oral Health, Taiwan)	Cross-sectional	*n* = 1,123; 19–40 y	Central adiposity, dyslipidemia (TG), and uric acid indicate metabolic-inflammatory burden promoting local inflammation.	Localized stage II/III periodontitis; waist circumference, TG, and uric acid positively associated with case status.	Mixed-age evidence; includes young adults, but applicability to adolescents is limited.	34605054
Nascimento et al. (46) (Pelotas 1982 Birth Cohort, Brazil)	Structural equation modeling (longitudinal cohort)	Exposure at 23 y; outcomes at 31 y	Obesity acts through metabolic-syndrome construct and systemic inflammation (CRP) to mediate periodontal damage.	Latent-variable analysis showed indirect positive path from adiposity via inflammation to periodontitis indicators.	Direct young-adult longitudinal evidence.	30447085
de Castilhos et al. (47) (Pelotas 1982 Cohort, Brazil)	Population-based cohort	*n* = 720; 24 y	Obesity associated with elevated CRP and poorer oral hygiene, enhancing gingival inflammation.	Obesity related to greater gingival bleeding (≥ 2 teeth) and calculus; no association with pockets.	Direct young-adult evidence.	22671969
Ekuni et al. (49) (Japan)	Cross-sectional (university students)	*n* = 618; 18–24 y	BMI reflects adiposity and increases host inflammatory load, impairing periodontal homeostasis.	CPI-defined periodontitis; 16 % higher odds per 1 kg/m^2^ BMI increase.	Direct young-adult evidence.	18942190
Ekuni et al. (48) (Japan)	Prospective cohort (students, entry → graduation)	*n* = 224; late teens–early 20s	BMI gain combined with poor oral hygiene exacerbates inflammation and tissue injury.	BMI increase linked to worse CPI; lack of interdental cleaning → higher BOP.	Direct young-adult evidence.	24813869
Franchini et al. (53) (Italy)	Case-control/cross-sectional (children)	*n* = 98; 10–17 y	Insulin resistance and poor hygiene amplify inflammatory response leading to gingivitis.	Overweight/obese children had higher gingival index; gingivitis more related to IR and hygiene than weight alone.	Direct adolescent evidence.	21793868
Iglesias Yunes et al. (55) (Brazil)	Cross-sectional (adolescents)	*n* = 109; 12–18 y	Increased adiposity induces low-grade inflammation and early periodontal tissue alterations.	Overweight/obese youth showed higher BOP, CPI codes 1 & 3, and incipient radiographic alveolar bone loss.	Direct adolescent evidence.	34937630
Ladeira et al. (57) (RPS Cohort, Brazil)	Structural equation modeling (population-based adolescents)	*n* = 2,515; 18–19 y	Insulin-resistance phenotype (TG/HDL, TyG, VLDL) and behavioral clustering increase chronic inflammatory load.	Latent “Chronic Oral Disease Burden” (BOP, PD ≥ 4 mm, CAL ≥ 3 mm, VPI ≥ 15 %) positively associated with obesity/IR and behaviors.	Direct late-adolescent/young-adult evidence.	37630703
Liu et al. (50) (NHANES 2011–2014, USA)	Cross-sectional (national survey)	*n* = 6,662; 30–44 y subgroup	General/central obesity elevates pro-inflammatory milieu and impairs vascular and repair functions.	Among 30–44 y adults, obesity remained associated with higher periodontitis prevalence after multivariable adjustment.	Indirect adult evidence; retained as contextual population-based evidence.	37935140

The target age group refers to adolescents and young adults. Mixed-age or adult studies were interpreted cautiously and used only to support biological plausibility or population-level context.

Mechanistically, excessive adiposity and IR is linked to a chronic low-grade inflammatory state in the body. Proinflammatory adipokines such as leptin and resistin rise markedly, while adiponectin—associated with healthy metabolism—decreases. TNF-α and IL-6 are further elevated, amplifying the body's response to infection (51). These mediators promote osteoclast differentiation, RANKL/OPG imbalance, and extracellular-matrix degradation, accelerating alveolar bone resorption. Concurrently, insulin resistance and hyperglycemia activate the AGE–RAGE axis, amplifying reactive-oxygen-species (ROS) production and endothelial dysfunction, leading to impaired microcirculation and delayed tissue repair (52). At the innate-immune level, obesity and IR impair neutrophil chemotaxis and phagocytosis, compromising bacterial clearance and sustaining chronic inflammation (54, 55). Conversely, periodontal infection and lipopolysaccharide (LPS) translocation into systemic circulation can enhance systemic inflammatory tone and interfere with insulin-signaling pathways, reinforcing a vicious bidirectional cycle between periodontal and metabolic disease (56, 57).

Collectively, these findings demonstrate that obesity and metabolic disorders are linked to periodontitis not merely through behavioral mechanisms. The two disorders interact bidirectionally via shared complex immuno-metabolic and vascular inflammatory pathways: systemic metabolic disturbance elevates inflammatory status to drive local periodontal damage, while periodontal inflammation further aggravates systemic metabolic dysfunction.

The association between obesity, metabolic dysfunction, and periodontal inflammation is supported by population-based studies and plausible immuno-metabolic mechanisms. However, many studies are cross-sectional, and confounding by oral hygiene, diet, smoking, and socioeconomic status cannot be fully excluded. Thus, causal direction and age-specific effects require further longitudinal confirmation.

### 3.4 Diet and behaviors

Based on current evidence, diet and health behaviors are closely linked to periodontitis and affect disease progression via distinct inflammatory and microbial pathways (Table 3). Using sugar intake as an example, among adolescents and young adults, higher consumption of added or total sugars is associated with greater gingival bleeding, increased probing depths, and worse composite periodontal outcomes (e.g., chronic oral disease burden, CODB); elevations in inflammatory markers such as IL-6 suggest involvement of low-grade systemic inflammation (58–60). Intake of ultra-processed foods (UPFs) is positively associated with moderate to severe periodontitis, potentially amplifying local immune responses via a “low fiber–additives–oral microbiome dysbiosis–systemic inflammation” pathway (61).

**Table 3:** The table summarizes studies on the impact of sugar intake and ultra-processed foods on periodontal health in youth. The table highlights the link between high sugar intake, dietary patterns, and periodontal damage, including increased bleeding on probing, clinical attachment loss, and periodontitis risk.

Evidence on the impact of sugar intake and ultra-processed foods on periodontal health in youth						
Study	Study design	Sample & age	Putative mechanism(s)	Key periodontal indicators/outcomes	Applicability to the target age group	PMID
Carmo et al. (58)	Cross-sectional, structural equation modeling	Public-school adolescents, Brazil (*n* = 405)	High added-sugar intake → low-grade systemic inflammation and biofilm dysbiosis contributing to a higher latent “chronic oral disease burden”	Composite burden including BOP and PD≥4 mm increased with added sugar (SEM paths significant).	Direct adolescent evidence.	29342369
Moreira et al. (59)	Cross-sectional (population-based)	Adolescents, 18–19 y, Brazil (*n* = 2,515)	Higher free/added sugar intake associated with biofilm-driven inflammatory response	Greater number of teeth with periodontal involvement (BOP, PD≥4 mm, CAL components) with high sugar exposure.	Direct late-adolescent/young-adult evidence.	32519237
Ladeira et al. (60)	Cross-sectional, structural equation modeling	Adolescents, 18–19 y, Brazil (*n* = 2,515)	Sugar intake above WHO/AHA recommendations → increased inflammatory oral-disease burden	Higher sugar intake associated with heavier chronic oral-disease burden (standardized coefficients significant).	Direct late-adolescent/young-adult evidence.	36504466
Cassiano et al. (61)	Cross-sectional (diet pattern by NOVA)	Young adults, Pelotas 1982 cohort, age 31 y (*n* = 537)	Ultra-processed foods (UPF) → dysbiosis/low-fiber, pro-inflammatory diet milieu	Moderate/severe periodontitis positively associated with UPF consumption (SEM coefficients significant)	Direct young-adult evidence.	36145111

The target age group refers to adolescents and young adults. The included studies primarily involved adolescent, late-adolescent, or young-adult populations.

Mechanistically, frequent consumption of fermentable sugars and acid loads maintains dental plaque at a persistently low pH, enriching acidogenic/aciduric taxa and fostering an inflammation-prone biofilm, thereby increasing the risk of deterioration in BOP, PPD and CAL (62). Low-fiber diets reduce masticatory stimulation and salivary buffering, undermining microbial homeostasis and mechanical self-cleaning. Sugar-sweetened beverages further exacerbate pathogenicity through rapid glycemic oscillations and local pH declines, heightening biofilm virulence and host inflammatory sensitivity (63). Conversely, systematic reviews and meta-analyses indicate that restricting free sugars may contribute to meaningful reductions in gingivitis, supporting the reversibility of a “lower sugar–lower inflammation” trajectory; exploratory randomized trials likewise observe additional decreases in bleeding indices alongside reductions in body weight and visceral adiposity, suggesting dual oral–metabolic benefits (64, 65). Overall, high sugar intake and UPF-dominated diets facilitate periodontal tissue destruction mainly by disrupting oral microbial equilibrium and activating persistent inflammatory responses.

Evidence linking high sugar intake, sugar-sweetened beverages, and ultra-processed foods to periodontal inflammation is suggestive but still limited. Most studies rely on self-reported diet and cross-sectional designs. Although short-term intervention studies support reduced gingival inflammation after sugar restriction, more age-specific longitudinal evidence is needed.

### 3.5 Plaque-retentive and iatrogenic factors

According to current evidence, plaque retention and iatrogenic factors are closely linked to the onset and progression of periodontitis (Table 4) (66, 67). Their common characteristic is the creation of a microenvironment that is “difficult to clean and permissive for biofilm maturation,” thereby exacerbating local inflammation and promoting CAL (68). Typical situations include over-contoured or overhanging restorations, subgingival or excessively deep crown margins, rough surfaces and microleakage, abnormal interproximal contacts, orthodontic attachments and fixed retainers, as well as steps or mismatches at implant-abutment junctions (69). These structures create “hiding spots” below the gumline, where biofilms, supported by low oxygen tension and abundant nutrients, accelerate the succession toward pathogenic microbiota, leading to increased BOP, PPD, and CAL (71).

**Table 4:** The table summarizes studies on the impact of plaque retention and iatrogenic factors on periodontal disease progression. The table highlights the effects of different retention methods and orthodontic treatments on gingival health, alignment stability, and periodontal outcomes such as gingival recession and clinical attachment loss.

Impact of plaque retention and iatrogenic factors on periodontal disease progression						
Study	Study design	Sample & age	Putative mechanism(s)	Key periodontal indicators/outcomes	Applicability to the target age group	PMID
Al-Moghrabi et al. (68)	Randomized controlled trial, 4-year follow-up	*n* = 42 at 4-year follow-up (21 fixed, 21 removable); mean age 21.15 ± 2.41 y.	Fixed retainers may better preserve alignment by reducing reliance on patient compliance, while both retainer types may hinder oral hygiene and increase plaque/gingival inflammation during long-term retention.	Fixed retainers showed better mandibular anterior alignment stability (lower irregularity) than vacuum-formed retainers at 4 years; no significant between-group differences were found for periodontal outcomes, and both groups showed common gingival inflammation and elevated plaque levels.	Direct young-adult evidence.	30075919
Petsos et al. (70)	Randomized clinical controlled trial, 12-month follow-up	n = 32 completed (15 fixed, 17 removable); mean age 16.1 ± 4.2 vs. 17.1 ± 5.4 y; non-smokers.	Fixed retainers may hinder interdental cleaning and promote plaque retention; removable retainers allow easier hygiene but rely on compliance.	No significant inter-group differences in REC or PPD over 12 months; gingival health at 12 months was 73.3% (fixed) vs. 88.2% (control), and recessions were clinically non-relevant.	Direct adolescent/young-adult evidence.	38355505
Celis et al. (71)	Ambispective observational study with untreated controls; ≥5-year post-treatment retention follow-up.	Treated n=89; controls n=88; age (treated): 11.92 y (T1), 14.26–15.14 y (T2), 19.37–20.18 y (T3); controls mean age 14.60 y.	Long-term fixed lingual retainers and retention-phase changes may increase susceptibility to gingival recession, especially in patients with thin phenotype and unfavorable skeletal/incisor-position patterns.	Recession incidence in treated patients increased from 11.24% at end of treatment (T2) to 67.42% at ≥5-year retention (T3); controls showed 15.91% prevalence. Risk indicators included female sex, thin phenotype, high mandibular plane angle, greater probing depth, and increased final lower-incisor inclination.	Direct adolescent-to-young-adult evidence.	39780360
Teubner et al. (72)	Retrospective cross-sectional radiographic study	*n* = 172 adolescents (86 treated, 86 controls); mean age 15.6 vs. 15.5 y (range 14.7–16.7 y).	Orthodontic bands may promote plaque retention and mechanical irritation, contributing to marginal bone changes during treatment.	Significant alveolar bone loss was observed on mesial surfaces of banded molars; distal surfaces showed no significant differences. A wider periodontal ligament space was found at the mesial right molar. Changes were minimal and not clinically significant.	Direct adolescent evidence.	29760824
Kredig et al. (74)	Prospective repeated-measures study	n = 50 adolescents; mean age 13.3 ± 1.8 y (age range 11–17 y)	Clear aligners may influence periodontal status via plaque retention at the gingival margin; host susceptibility assessed using IL-1 polymorphism and inflammatory biomarkers (aMMP-8, marker pathogens).	No significant increase in periodontal inflammation or aMMP-8 over treatment; red/orange complex pathogens stayed low, while Capnocytophaga spp. and Fusobacterium spp. changed significantly; GI correlated with aMMP-8, and genotype showed no effect on GI.	Direct adolescent evidence.	40042542
Adanur-Atmaca et al. (75)	Randomized controlled trial (1-year)	*n* = 132 orthodontic patients; median age 16 y	Retainer material and surface characteristics may influence plaque retention, gingival inflammation, and calculus accumulation.	Memotain showed lower gingival and calculus indices; no clinically significant periodontal deterioration or relapse after 1 year.	Direct adolescent evidence.	33587126

The target age group refers to adolescents and young adults. Most studies in this table directly involved adolescent or young-adult orthodontic populations.

Mechanistically: (1) Increased surface roughness enhances initial bacterial adhesion and promotes subsequent bacterial proliferation (73); (2) microleakage and degradation products from dental materials stimulate sulcular inflammation, upregulating IL-1β, TNF-α, and IL-6, and driving matrix degradation via matrix metalloproteinases (MMPs) (74); (3) biofilm-host interactions promote vasodilation, disruption of the epithelial barrier, and enlargement of ulcerated areas, facilitating deeper penetration of bacteria and lipopolysaccharides (LPS) into connective tissue (76); (4) restricted access for cleaning—compounded by patient-level technique and adherence issues—results in insufficient mechanical debridement and a self-reinforcing inflammatory loop (77). Therefore, eliminating anatomical and restoration-related microenvironments that promote plaque retention helps interrupt persistent local inflammatory cascades and is a key component in the prevention and clinical management of periodontitis.

Plaque-retentive and iatrogenic factors have strong local biological plausibility, as they directly promote biofilm accumulation. However, clinical evidence is heterogeneous because studies differ in appliance type, restoration quality, follow-up time, and oral hygiene control. Their effects should therefore be interpreted as modifiable local risk enhancers rather than deterministic causes.

## 4 Discussion

This review examines whether periodontitis displays a younger onset trend in the general population, finds that early periodontal burden is more concentrated in subgroups exposed to modifiable risk factors, and further elaborates how such risk factors affect the onset and progression of periodontitis via relevant inflammatory pathways (78). Within these subgroups, multiple pathways may act additively, and in some cases synergistically, to accelerate the progression of periodontitis. Tobacco and e-cigarette exposure may impair host defenses through vascular and immune modulation, oxidative stress, and microbiome remodeling, and may also reduce treatment responsiveness, thereby promoting the development of periodontitis (79, 80). In adolescents and young adults, the periodontal implications of tobacco and e-cigarette exposure should be interpreted in the context of age-specific exposure and behavioral patterns. Unlike older adults, younger individuals often have shorter cumulative exposure and less severe baseline periodontal destruction; however, nicotine exposure may begin during a critical developmental period and occur through intermittent or social smoking, flavored e-cigarette experimentation, and dual use of combustible cigarettes and ENDS. These behaviors may also cluster with inadequate oral hygiene, irregular dental attendance, alcohol consumption, high sugar intake, sleep irregularity, and lower perception of oral-health risks, thereby increasing susceptibility to early periodontal inflammation. Moreover, smoking- and vaping-related biological changes, including altered neutrophil function, cytokine dysregulation, oxidative stress, vascular changes, and dysbiotic shifts in the subgingival microbiome, may precede overt clinical attachment loss in younger populations. Therefore, findings from adult or mixed-age studies should not be directly extrapolated to adolescents and young adults, and future longitudinal studies focusing specifically on early initiation, cumulative exposure, dual use, and dose–response relationships are needed. Obesity and metabolic dysfunction may amplify inflammation through adipokine imbalance and the AGEs–RAGE–ROS axis while also impairing periodontal tissue repair. In addition, bidirectional associations between these conditions and periodontitis have been reported, with worsening periodontal inflammation potentially further contributing to obesity and metabolic dysfunction (81). Diets high in sugar and ultra-processed foods (UPFs) may sustain a low-pH, inflammation-prone biofilm environment and aggravate periodontal inflammation, whereas intervention studies on sugar reduction suggest that gingivitis is at least partly reversible (82). Plaque-retentive and iatrogenic factors may further promote pathogenic biofilm niches through morphological irregularities, surface roughness, and microleakage, particularly when oral hygiene performance and treatment adherence are poor. Therefore, rather than relying solely on post-onset treatment, reducing harmful exposures and improving plaque control in the pre-disease stage can bring greater benefits to long-term periodontal health. Such measures include enhancing oral health literacy, promoting effective self-care, and delivering sustained preventive support (83).

These modifiable risk factors further highlight the importance of timely identification and early intervention, including early screening for bleeding on probing (BOP) and calculus, risk-stratified recall, and the identification, documentation, and targeted intervention for high-risk individuals through school-based and primary-care programs. To routinize these measures, preventive efforts should be embedded within existing school health, primary health care, and adolescent health service systems, while also being integrated with non-communicable disease prevention and control. Specifically, such strategies should be advanced in a coordinated manner across multiple levels. First, reducing exposure to conventional tobacco and electronic nicotine delivery systems (ENDS) among adolescents and young adults should be a key component of prevention. This may involve comprehensive measures such as tax and price policies, marketing restrictions, smoke-free school environments, age verification, and cessation support, which together may reduce the accessibility and social acceptability of tobacco and e-cigarette products, thereby lowering their adverse impacts on periodontal inflammation and host immune response. Second, in terms of improving the food environment, measures such as establishing nutritional standards for school canteens, restricting the marketing of high-sugar products to minors, implementing front-of-pack nutrition labelling (FOPL), a simplified nutrition information system displayed on the front of food packages, and reducing the availability of sugar-sweetened beverages may facilitate healthier dietary choices and reduce the sustained contribution of high-sugar and ultra-processed diets to dysbiotic biofilm ecology, inflammatory burden, and metabolic disturbances. At the same time, greater emphasis should be placed on service integration by incorporating brief behavioral counseling, standardized screening for early inflammatory indicators, and clear referral pathways into school health services, primary care, and adolescent health care, thereby improving the accessibility and continuity of risk identification and early intervention. For adolescents with obesity or metabolic abnormalities, comprehensive management should include oral health assessment, lifestyle counseling, necessary specialist referral, ongoing monitoring, scheduled recall, and the establishment of individualized health records, in order to better address the potentially bidirectional relationship between periodontitis and systemic metabolic dysfunction. Plaque-retentive and iatrogenic factors may be addressed through quality standards for restorations and orthodontic appliances, regular maintenance follow-up, enhanced patient education to strengthen awareness of self-performed oral care, and targeted professional training for clinicians, particularly primary-care orthodontic providers. Looking forward, chairside biomarkers, salivary diagnostics, and digital health tools may facilitate earlier and more precise identification and monitoring of disease risk.

Overall, the available evidence supports the view that the periodontal disease burden in adolescents and young adults tends to be concentrated in high-risk subgroups. This pattern suggests that the early emergence of periodontitis in this population is more closely related to the clustering of modifiable risk factors. Accordingly, preventive strategies should shift from passive post-disease treatment toward earlier and more comprehensive proactive risk management, integrating risk reduction, early screening, risk-stratified follow-up, long-term maintenance, and relapse prevention. Such strategies should be implemented through existing school health, primary health care, and adolescent health service systems, in coordination with non-communicable disease prevention and control. Given that periodontal health is associated with multiple systemic health conditions, particularly with well-documented bidirectional links to diabetes and cardiovascular disease (84, 85), and potential correlations with certain neurodegenerative diseases and malignancies (86), delaying early periodontal progression and improving periodontal health in adolescents and young adults carries not only oral health significance but also broad public health implications, and can positively shape their long-term quality of life and health outcomes.

## 5 Limitations

This review should be interpreted in light of several limitations. As a narrative review, it provides an integrative evidence synthesis and a prevention-oriented interpretive framework rather than a quantitative pooled estimate. The included literature is highly heterogeneous in outcome definitions, diagnostic criteria, and examination methods, which limits direct comparability across studies. In adolescents and young adults, most available evidence is cross-sectional, thereby constraining causal inference and temporal interpretation of progression along the gingivitis-to-periodontitis continuum. In particular, residual confounding by oral hygiene behaviors, socioeconomic status, diet, smoking, obesity, access to dental care, and health literacy may influence the observed associations across different exposure groups. In addition, some evidence supporting specific risk pathways is derived from mixed-age populations and may not be fully generalizable to this population. Although the Line A/Line B framework improves interpretive clarity, outcome mapping remains dependent on the reporting quality and definitions used in the primary studies. Future research should further prioritize age-stratified studies in this group, with greater consistency in the definition and measurement of early periodontal indicators and established disease outcomes, to enhance comparability across studies and improve the interpretation of the periodontal disease continuum.
